# The intervention dilemma and high burden of children with autism in Guizhou province, Southwest China

**DOI:** 10.3389/fpsyt.2022.929833

**Published:** 2022-11-02

**Authors:** Ye Liu, Fang Zhou, Jixuan Qin, Yong Lin, Tonghuan Li, Chengyong Zhu, Fang Long, Xike Wang, Xiao Hu, Hao Zhou

**Affiliations:** ^1^Department of Otolaryngology, Guizhou Provincial People’s Hospital, Medical College of Guizhou University, Guiyang, China; ^2^Department of Developmental Behavioral Pediatrics, Guizhou Provincial People’s Hospital, Medical College of Guizhou University, Guiyang, China; ^3^Department of Pediatric Rehabilitation, Anshun Women’s & Children’s Hospital, Anshun, China; ^4^Department of Pediatric Rehabilitation, The First People’s Hospital of Zunyi, Zunyi, China; ^5^Department of Pediatric Rehabilitation, Affiliated Hospital of Zunyi Medical University, Zunyi, China; ^6^Department of Pediatric Rehabilitation, The Third Affiliated Hospital of Guizhou Medical University, Duyun, China; ^7^Department of Healthcare, Liupanshui Women & Children Hospital, Liupanshui, China; ^8^Department of Neurology, Guizhou Provincial People’s Hospital, Guiyang, China

**Keywords:** autism, rehabilitation status, disease burden, questionnaire, China

## Abstract

**Background:**

Autism spectrum disorder (ASD) is a highly disabling neurodevelopmental disorder, and the burden is high. Data on the burden of ASD are limited in China, especially in the southwest. Therefore, the aims of this study were to investigate the intervention status and burden of children with ASD in Southwest China.

**Materials and methods:**

Families of children with ASD were recruited from hospitals, special education schools, and private rehabilitation centers; they participated in the survey and completed the questionnaire. Descriptive analysis was conducted on the questionnaire results, which included basic demographic characteristics, rehabilitation status, and burden. Multivariate analysis was used to analyze the association of basic family demographic characteristics, rehabilitation status, and costs of ASD.

**Results:**

A total of 231 families of children with ASD participated in this survey, and 78.35% (181/231) of the children with ASD were male. The mean age was 4.34 ± 2.09 years. A total of 55.84% (129/231) of the children with ASD had an intellectual disability. Only 46.32% (107/231) started receiving intervention within 1 month after diagnosis. The institutions for rehabilitation interventions for children with ASD were mainly tertiary hospitals (39.39%), special education schools (29.87%) and private rehabilitation institutions (21.64%). For a total of 42.86% (99/231) of the children with ASD, the duration of the intervention was less than 10 h per week. A total of 74.89% (173/231) of the children with ASD received a rehabilitation intervention at home. A total of 66.67% of the parents were satisfied with the treatment. The monthly cost of medical intervention for the patients of children with autism was 7,225 ± 474 RMB ($1,134 ± 74), and the non-medical intervention cost was 2,133 ± 107 RMB ($334 ± 17). The annual burden of patients with autism was 86,700 ± 5,688 RMB ($13,596 ± 892). The estimated total annual burden of ASD was 5.548 billion RMB ($870 million) in Guizhou province.

**Conclusion:**

The results revealed that rehabilitation resources are limited and that the burden of ASD is high in Guizhou province; therefore, improving the rehabilitation status and easing the burden of children with ASD is urgent in these regions.

## Introduction

Autism spectrum disorder (ASD) is a group of highly heterogeneous neurodevelopmental disorders (1). ASD has been associated with significant social functional impairments and lasts a lifetime, and it can severely impact the quality of life of patients with ASD and their families (2). The number of individuals diagnosed with ASD has obviously increased in recent decades. The prevalence of ASD was 1/44 in the U.S. and our previous national epidemiological study showed that the prevalence of ASD was 0.7% in the Chinese child population (3, 4). According to preliminary estimates, there are at least millions of patients with ASD, and an epidemiological survey revealed that ASD is the main cause of disability in children under the age of 6 in China (5, 6). Accurate estimates of the economic effect of ASD are very important and urgently needed to illustrate the responsibilities of the families of children with ASD and society.

The burden of ASD is very high, and the total burden includes direct medical costs (medical checkup costs, rehabilitation costs, etc.), and indirect costs (family care, education, living expenses, etc.) (7); however, the overall and economic effects of ASD are not well-established worldwide. A previous study indicated that the lifespan costs of individuals with ASD with or without intellectual disability were $2.4 and $1.4 million in the United States and $2.2 and $1.4 million in the United Kingdom, respectively (8). Hong et al. reported that the economic burden of ASD was estimated to be $2,700,596 in 2008 and $9,645,503 in 2015 in South Korea (9). Another study showed that ASD placed a higher burden on caregivers compared with other mental illnesses, such as schizophrenia (10). Currently, the data on ASD burden mainly come from developed countries, while such data are rare in developing countries. The limited data demonstrated that the burden of children with autism was 19,582.4 RMB ($3,704) per year in Beijing City, China (11). Another study showed that the average loss of annual income associated with having a child with ASD was 44,077 RMB ($7,226) in Changsha City, China (12). Medical resources and economic levels are uneven in China (13), and Beijing and Changsha are developed cities. Our previous research found low levels of awareness and knowledge of ASD among child health care workers in Guizhou province in China (14), which is an underdeveloped region in terms of medical resources and economic level. Therefore, investigating the rehabilitation status and burden of children with ASD is urgent in these regions, and the aim of this study was to focus on this issue.

## Materials and methods

### Study sites and participants

The study was conducted from March 2022 to April 2022 in Guizhou province, China, which is located at 24°37’–29°13’N, 103°36’–109°35’E. The resident population of Guizhou in 2021 was 38,562,100 according to the 7th National Census,^1^ and the GDP was ranked 22 among the 31 provinces in mainland China, which is a relatively low level within China. Medical resources, especially rehabilitation resources for ASD, are insufficient in Guizhou province, and only a few hospitals have rehabilitation centers for children or developmental behavioral pediatrics units. Thus, families of children with ASD were recruited from six rehabilitation centers for children (Guizhou Provincial People’s Hospital, Anshun Women’s & Children’s Hospital, the First People’s Hospital of Zunyi, the Affiliated Hospital of Zunyi Medical University, and Liupanshui Women’s & Children’s Hospital), two special educational institutions (Shanchen and Huanlechuan Special Educational Institutions, which are representative special educational institutions for children with ASD in Guizhou province), and a community hospital (Shuikoushi Community Hospital, which is the only community hospital with a training center for ASD in Guizhou province).

### Questionnaire

The information was collected by using our self-designed questionnaire. The multidisciplinary team for questionnaire development was composed of clinical experts with rich experience in ASD, epidemiologists, special education teachers, and public health experts. All items of the questionnaire were original, and the questions were arranged in the questionnaire according to the expert group’s recommendations. After designing the questionnaire, we conducted a pilot study to evaluate the content validity of the questionnaire and invited 30 families of children with ASD from Guizhou Provincial People’s Hospital to complete the questionnaire. All feedback indicated that the questions were easy to understand in the Chinese cultural context.

The questionnaire consisted of three parts. The first part collected the basic demographic characteristics of the families of children with ASD, including the child’s age at the time of the study and sex; parents’ education levels, occupations, and economic income; parents’ understanding of their child’s developmental age according to his or her abnormal developmental trajectory; age of the child at the time of ASD diagnosis; and whether the child had an intellectual disability (note: the cognitive level was based on the results of cognitive tests such as the Gesell assessment). The second part contained nine items to assess rehabilitation training status, including the interval from diagnosis to receiving rehabilitation training, where a patient received rehabilitation training, whether the parents received family intervention training, whether the patient received a family intervention, and the employment situation of the parents. The third part contained six questions that evaluated the burden of ASD, including monthly out-of-pocket payments for rehabilitation training, disability benefits, other non-medical expenses (the costs of accommodations, meals, transportation), the share of rehabilitation training costs in the household income, and whether rehabilitation training had to be given up due to high health care costs. All questions were multiple choice and included options for “Yes” and “No.”

### Data collection

WeChat (Tencent Corp) is the most popular social software used in China. The questionnaires were developed and sent through the WeChat network by doctors and special education teachers using Sojump (Changsha ran Xing InfoTech Ltd., Changsha, China), which is a professional online survey, evaluation and polling platform that provides personalized services, including questionnaire design and data collection. The families of children with ASD used a mobile phone to scan a QR code, and there was no time limit to complete the questionnaire. If participants had any questions, professional staff answered them. All participants voluntarily and anonymously completed the questionnaires and provided oral informed consent. Questionnaires with incomplete basic information and unchecked questions were excluded.

### Data analysis

Data analysis was performed by utilizing SPSS 26.0 (SPSS Inc., Chicago, IL). Enumeration data are described as the mean ± SD, and categorical variables are presented as percentages. The basic information, the status of training, and the costs were analyzed by descriptive analysis. The total annual burden of ASD was estimated based on the total number of children with ASD and the average cost per person every year in Guizhou province. The total number of children with ASD in Guizhou province was calculated according to an ASD prevalence of 0.7% in the Chinese population and a total of 9,200,000 children aged 0–14 years (contents refer to the 7th National Census (see text footnote 1) in Guizhou province. A total of approximately 64,000 (0.007 × 9.2 million) children had been diagnosed with ASD. The effects of the different variables on the therapeutic effect and costs were compared by the chi-square test, and the Friedman test was conducted to explore the contributions of the variables to the therapeutic effect and costs. All tests were two-tailed, and a *P*-value of less than 0.05 was considered statistically significant.

## Results

### Basic information of participants

A total of 231 families of children with ASD participated in this survey; 78.35% (181/231) of the children with ASD were male, and 21.65% (50/231) were female. The mean age of the children with ASD was 4.34 ± 2.09 years. The parents reported that the mean developmental age of their children with abnormal developmental trajectories was 2.11 ± 1.09, and the mean age at the time of ASD diagnosis was 2.58 ± 1.35. A total of 51.51% (119/231) of the families earned less than 5,000 RMB ($786) per month. The main caregivers in rehabilitation training for the children were their mothers. A total of 55.84% (129/231) of the parents reported that their child with ASD had an intellectual disability (Table 1).

**TABLE 1:** Basic information of respondents (*n*, %).

Characteristics	Total (*n*)	Ratio (%)
Number	231	
**Age (years, mean ± mean)**		
Present age	4.34 ± 2.09	
Children with abnormal developmental trajectories	2.11 ± 1.09	
At diagnosis with ASD	2.58 ± 1.35	
**Sex**		
Male	181	78.35
Female	50	21.65
**Father’s career**		
Professional	53	22.94
Civil servant	19	8.22
Farmer	42	18.18
Freelancer	117	50.66
**Mother’s career**		
Professional	50	21.65
Civil servant	10	4.32
Farmer	50	21.65
Freelancer	121	52.38
**Father’s level of education**		
Junior	156	67.53
Undergraduate	64	27.71
Graduate	11	4.76
**Mother’s level of education**		
Junior	152	65.80
Undergraduate	73	31.60
Graduate	6	2.60
**Monthly income**		
<5,000 RMB ($786)	119	51.51
5,000–10,000 RMB ($786–1,572)	94	40.69
>10,000 RMB ($1,572)	18	7.80
**Caregiver in rehabilitation training**		
Father	25	10.82
Mother	145	62.77
Grandparent	59	25.54
Nanny	2	0.87
**Developmental delay**		
Yes	129	55.84
No	102	44.16

### Intervention status of children with autism spectrum disorder

Among the children with ASD who participated in the questionnaire, only 46.32% (107/231) started an intervention within 1 month after diagnosis. The institutions for rehabilitation interventions for the children with ASD were mainly tertiary hospitals (39.39%), special education schools (29.87%) and private rehabilitation institutions (21.64%). A total of 42.86% (99/231) of the children with ASD had an intervention duration of less than 10 h per week, and only 9.53% (22/231) had an intervention duration of more than 30 h per week. More than 50% of the children with ASD received family intervention training, and 74.89% (173/231) received rehabilitation intervention at home. A total of 22.08% of the children with ASD received other non-behavioral intervention therapies, such as acupuncture, mouse nerve growth factor treatment, and hyperbaric oxygen treatment. A total of 66.67% of the parents were satisfied with the treatment, and almost two-thirds of the families of children with ASD chose to resign from their jobs after their children were diagnosed with ASD (Table 2).

**TABLE 2:** The rehabilitation intervention status of children with autism spectrum disorder (ASD) in Guizhou province (*n*, %).

Characteristics	Total	Ratio (%)
Number	231	
**Interval from diagnosis to receiving intervention**		
<1 month	107	46.32
1–3 months	52	22.51
3–6 months	30	12.99
>6 months	42	18.18
**Intervention site**		
Tertiary hospital	91	39.39
Special education school	69	29.87
Private rehabilitation institution	50	21.64
Community hospital	21	9.09
**Total intervention time per week**		
<10 h	99	42.86
10–19 h	75	32.46
20–29 h	35	15.15
>30 h	22	9.53
**Receiving family intervention training**		
Yes	126	54.55
No	105	45.45
**Family intervention program**		
Yes	173	74.89
No	58	25.11
**Treatment beyond intervention**		
Yes	51	22.08
No	180	77.92
**Treatment effect**		
Satisfied	154	66.67
Unsatisfied	77	33.33
**Parent resignation from employment**		
Father	17	7.36
Mother	104	45.02
Both	12	5.20
None	98	42.42

### Costs for families of children with autism spectrum disorder

The monthly cost of medical intervention for patients with autism was 7,225 ± 474 RMB ($1,134 ± 74), of which the self-funded portion was 3,679 ± 466 RMB ($577 ± 73), and the subsidy of the China Disabled Persons’ Federation (CDPF) was 1,321 ± 82 RMB ($207 ± 13). Notably, more than 70% of the families were aware of the CDPF subsidy policy and received funding. The non-medical intervention cost was 2,133 ± 107 RMB ($334 ± 17). The annual burden of patients with autism was 86,700 ± 5,688 RMB ($13,596 ± 892). Approximately 30% of the parents thought that the burden accounted for 70% of their family income, and 37.66% of them had planned to give up treatment because of the heavy burden.

The total annual burden of ASD was estimated based on the total number of children with ASD and the average cost per person every year in Guizhou province. The total expenses were 5.548 billion RMB (64,000 × 86,700 RMB, equivalent to US $870 million), the total annual medical expenses were 3.9 billion RMB (64,000× 7,225 × 12 RMB, equivalent to US $610 million), and the total annual non-medical expenses were 1.657 billion RMB (64,000 × 2,133 × 12 RMB, equivalent to US $260 million) in Guizhou province.

### Impacts of the basic demographic characteristics on training and costs of autism spectrum disorder

**FIGURE 1:**
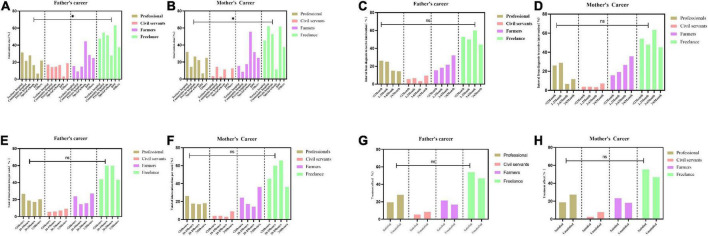
Influence of parental occupation on the intervention status of children with autism spectrum disorder (ASD), **(A,B)** for the choice of rehabilitation institutions, **(C,D)** for the interval from diagnosis to receiving intervention, **(E,F)** for the total duration of the intervention per week, **(G,H)** for satisfaction with the intervention effect. *p < 0.05; ns, *p* > 0.05.

There were significant differences in the choice of rehabilitation institutions among parents of different occupations; for example, farming families preferred to choose special education schools for their children’s ASD training, and professional and civil servants always preferred tertiary hospitals or private rehabilitation institutions (Figures 1A,B). There was no significant difference among children with ASD in the interval from diagnosis to receiving intervention, the total duration of the intervention per week, or the satisfaction with the intervention effect by parents’ occupation (Figures 1C–H).

**FIGURE 2:**
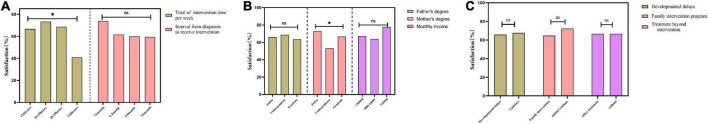
Analysis of influencing factors of satisfaction with intervention treatment for children with Autism spectrum disorder (ASD). **(A)** For total intervention duration per week and interval from diagnosis to receiving intervention, **(B)** for parents’ degree and monthly income, and **(C)** for developmental delays, family intervention program, and treatment beyond intervention. *p < 0.05; ns, *p* > 0.05.

From Figures 2A–C we can see that the intervention effect of children with ASD was associated with the total intervention duration per week, and families with 10–19 h of intervention had the highest satisfaction with the treatment effect (*P* < 0.05). There were significant differences in satisfaction with the intervention treatment for children with ASD among mothers with different educational backgrounds. Mothers with a junior college degree or below had the highest satisfaction (*P* < 0.05). Other factors, such as paternal educational background, parents’ occupations, monthly family income, whether there was a developmental lag, whether children received treatment other than the rehabilitation intervention, and whether family intervention was carried out, had no significant impact on satisfaction with the intervention effect for children with ASD.

**FIGURE 3:**
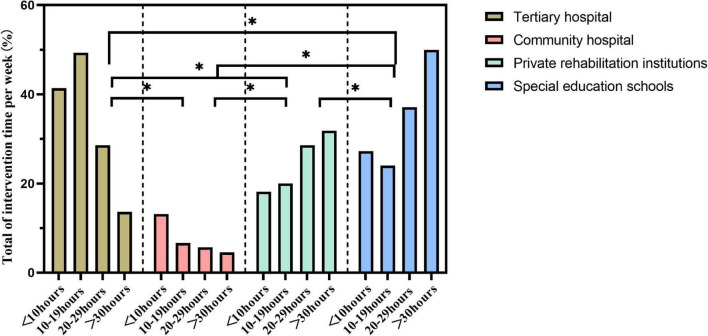
Influence of rehabilitation location on the total weekly intervention duration for children with Autism spectrum disorder (ASD). *p < 0.05; ns, *p* > 0.05.

The weekly intervention duration for children with ASD was significantly different among the different rehabilitation sites (*P* < 0.05), with durations of more than 30 h for private rehabilitation institutions and special schools and less than 10 h for public hospitals (Figure 3).

**FIGURE 4:**
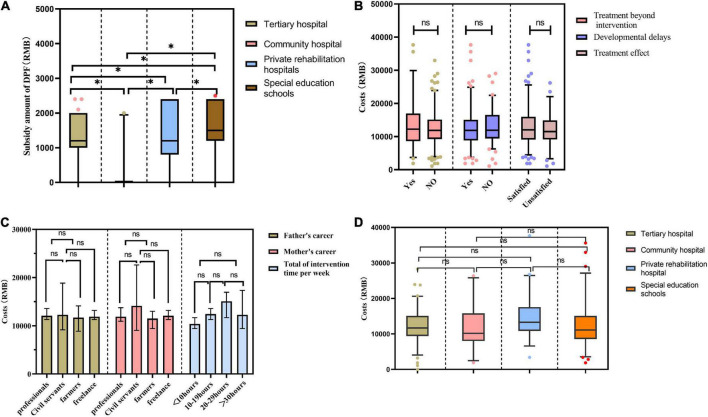
Impacts of different factors on rehabilitation costs. *p < 0.05; ns, *p* > 0.05.

The DPF subsidy for children with ASD varied significantly among different rehabilitation treatment sites. The highest subsidy was 1,863 ± 110 RMB and 1,825 ± 245 RMB per month in tertiary hospitals and special education schools, respectively, and the lowest was in community rehabilitation institutions (Figure 4A). There was no significant difference between the costs and parents’ occupations, the total duration of the intervention per week, the place where intervention treatment was received and whether children received other treatments (Figures 4B–D).

## Discussion

Autism spectrum disorder is the most common highly disabling neurodevelopmental disorder in childhood and has a high burden. The exact costs of ASD worldwide are still unclear. In this study, we first described the intervention status and burden of children with ASD in Guizhou province, Southwest China. The cross-sectional survey revealed that only 46.32% of the children with ASD started an intervention within 1 month after diagnosis. The institutions for rehabilitation interventions for children with ASD were mainly tertiary hospitals, special education schools and private rehabilitation institutions. For almost half of the children with ASD, the duration of intervention was less than 10 h per week. A total of 74.89% of the children with ASD received a rehabilitation intervention at home. Only two-thirds of the parents were satisfied with the treatment. In families of children with ASD with a high burden, the annual burden of patients with autism could reach as high as 86,700 ± 5,688 RMB ($13,596 ± 892).

### Intervention status of autism spectrum disorder

The results of this survey showed that the parents realized that the mean developmental age of their children with abnormal developmental trajectories was 2 years, and the mean age at the time of ASD diagnosis was 2.5 years. Only half of the children with ASD started an intervention within 1 month after diagnosis, indicating that many children with ASD received interventions around the age of 3 years. This seems to be a challenge for ASD prognosis, and a previous study demonstrated that early screening and intervention were extremely important for ASD prognosis (15). Early ASD screening networks have been established in many developed cities in China, such as Shanghai, Tianjin, and Guangzhou (16–18). Medical resources and economic levels are unevenly distributed in China (13). Guizhou province is located in Southwest China, which is an underdeveloped region in terms of medical resources and economic level. Early screening of ASD is limited, and our previous research found low levels of awareness and knowledge of ASD among child health care workers in Guizhou province (14). Thus, there is an urgent need to establish the early screening network of ASD in these less developed areas, in China.

In addition to the delay in the age of ASD screening and intervention, the results of this study revealed that ASD public medical resources are insufficient in Guizhou province. Half of the families of children with ASD chose special education schools and private rehabilitation institutions to conduct interventions. A previous investigation showed that ASD interventions mainly occur in private institutions in China (19). It is interesting to note that parents’ occupations impact the choice of rehabilitation institutions, and the main reason is that family income may affect the choice of rehabilitation institutions and family income varies across different occupations. To address this dilemma of a medically underserved population, in recent years, experts have called for enhanced family intervention for children with ASD, and the family may play a key role in ASD intervention and provide a clear benefit for ASD prognosis (20–22). In this study, a total of 74.89% of the children with ASD received rehabilitation interventions at home, which revealed that family intervention has become increasingly valued. However, while many children with ASD received family intervention, for almost half of the children with ASD, the duration of the intervention was less than 10 h per week, especially for those receiving interventions in public hospitals. This may be related to medical students unwillingly to be occupied in pediatrics in China (23, 24) and hospitals being short-staffed for the provision of ASD interventions. Of concern, 22.08% of the children with ASD received other non-behavioral intervention therapies, such as acupuncture, mouse nerve growth factor treatment, and hyperbaric oxygen treatment; however, these therapies lack evidence-based recommendations.

### Costs of autism spectrum disorder

The results of this study showed that the annual burden of patients with autism could be as high as 86,700 ± 5,688 RMB ($13,596 ± 892) in Guizhou province, China. In 2011, Xiong et al. reported that the burden of children with autism was 19,582.4 RMB ($3,704) per year in Beijing City, China (11). In 2015, Ou et al. conducted a survey and revealed that the average loss of annual income associated with having a child with ASD was 44,077 RMB ($7,226) in Changsha City, China (12). We can see from the current study’s data that the cost of ASD may have increased in China over time, which is similar to a previous study showing that the economic burden of ASD in 2015 was quadruple that in 2008 in South Korea (9). Seventy percent of the families of children with ASD received DPF funding in our study; however, ASD costs were mainly self-funded. The burden accounted for 70% of the families’ income, and approximately one-third of the families planned to give up treatment because of the heavy burden. The results indicated that the burden of ASD is very high for families of children with ASD and the government in China and that the insurance system for ASD needs to be further improved. The results illustrated that the highest DPF subsidy was for special education schools, which can explain why farming families preferred to choose special education schools for their children’s ASD training. It is worth noting that the burden of ASD comorbid with other diseases was obviously increased in a previous study (8); however, whether the children had developmental delays was not related to the costs of ASD in our study. It could be that the respondents were younger in this study, and other costs, such as educational expenses, had not been incurred.

### Limitations

This is the first study to describe the intervention status and burden of children with ASD in Guizhou province, Southwest China. However, there are two limitations in this survey. First, the sample size was relatively small, and the survey was conducted mainly in cities, making some of the results prone to bias. Second, the respondents were younger in this study, and other costs had not been incurred, so the actual burden may have been underestimated here. Therefore, a multicenter, multidimensional survey is urgently needed in future studies.

## Conclusion

In conclusion, this is the first study to describe the intervention status and burden of children with ASD in an underdeveloped city in China. The results revealed that the rehabilitation resources were limited and that the burden of ASD was high in Guizhou province; therefore, improving the rehabilitation status and easing the burden of children with ASD is urgent in these regions.

## Data availability statement

The original contributions presented in this study are included in the article/supplementary material, further inquiries can be directed to the corresponding authors.

## Ethics statement

The studies involving human participants were reviewed and approved by the Guizhou Provincial People’s Hospital. Written informed consent to participate in this study was provided by the participants’ legal guardian/next of kin.

## Author contributions

HZ and XH conceived the study. YeL and FZ contributed to the analysis, synthesis, and interpretation of the results and wrote the manuscript. JQ, YoL, TL, CZ, and FL contributed to the data collection. All authors contributed to the preparation of the manuscript.

## Footnotes

1
http://www.stats.gov.cn/tjsj/pcsj/rkpc/d7c/202111/P020211126523667366751.pdf
